# Dyadic coping experiences of young and middle-aged patients undergoing PCI for coronary heart disease and their spouses during the perioperative period: a qualitative study

**DOI:** 10.3389/fcvm.2026.1791315

**Published:** 2026-06-04

**Authors:** Suwen Ma, Shuyu Lu, Hua Lu, Hong Li, Yi Liang, Juanjuan Liu, Pinyue Tao

**Affiliations:** 1 Department of Anaesthesiology, The Second Affiliated Hospital of Guangxi Medical University, Nanning, Guangxi, China; 2 Heart Center, Guilin People's Hospital, Guilin, Guangxi, China

**Keywords:** coronary heart disease, dyadic coping, PCI perioperative period, qualitative research, spouse

## Abstract

**Objective:**

This study focuses on the dyadic coping units formed by middle-aged and young patients and their spouses during the perioperative period of percutaneous coronary intervention(PCI) given the trend toward a younger onset of coronary heart disease. It explores their experiences of dyadic coping during this critical transitional phase.

**Methods:**

Based on the theory of dyadic illness management, this descriptive qualitative study involved semistructured joint interviews with 19 pairs of middle-aged and young PCI patients and their spouses. Content analysis was used to identify themes, and the study adhered to the COREQ reporting guidelines.

**Results:**

The study identified five key themes: differing coping styles during the decision-making phase regarding stents, the exacerbation of psychological stress due to dual financial pressures, conflicting patterns of spousal interaction, variations in patients’ perceptions of spousal support, and a lack of clear understanding regarding long-term disease management.

**Conclusion:**

Middle-aged and young patients with coronary heart disease and their spouses face multiple challenges during the perioperative period of PCI. It is recommended that a dyadic, collaborative care intervention program be developed at the family level that integrates health literacy enhancement, financial support, spousal communication and information-based continuity of care to improve disease management outcomes.

## 1 Introduction

Coronary heart disease (CHD) is among the leading causes of death and disability worldwide (1). With socioeconomic development and lifestyle changes, the prevalence of CHD continues to increase, and the age of onset is trending toward younger individuals (2). As the core treatment for CHD, percutaneous coronary intervention (PCI) effectively relieves symptoms, saves lives, and facilitates early social reintegration. However, it is not a curative intervention; patients require long-term adherence to medication, risk factor management, and lifestyle modifications postprocedure (3). Unfortunately, young and middle-aged patients often neglect long-term self-management because of heavy work pressure, family responsibilities, or an insufficient awareness of disease severity, leading to increased risks of recurrent cardiovascular events. In this context, as patients' closest caregivers and sources of emotional support, spouses play a critical role in chronic disease management. Dyadic coping (DC) refers to joint coping strategies adopted by partners through communication, support, and collaboration when they face shared stressors (4). Research has indicated that joint participation in disease management by both spouses not only enhances patient treatment adherence and quality of life but also strengthens marital intimacy (5). The theory of dyadic illness management (TDIM) (6) emphasizes the dynamic process of patients and caregivers collaboratively managing illness as a “team” and its impact on health. The PCI perioperative period represents a critical transition phase for middle-aged and young patients, during which they shift from acute treatment to long-term chronic disease management. The quality of dyadic coping during this period directly influences the subsequent establishment of self-management behaviors. Current research predominantly employs quantitative methods, and an in-depth exploration of spousal interaction experiences within cultural contexts is lacking (7, 8). Given the dynamic and culturally specific nature of dyadic coping, this study, which is grounded in the TDIM, adopts a descriptive qualitative approach to investigate in depth the authentic experiences of middle-aged and young patients undergoing PCI and their spouses during the PCI perioperative period. It aims to reveal the interactive characteristics and challenges of their dyadic coping, thus providing a basis for developing dyadic collaborative care interventions tailored to the local cultural context.

## 2 Article type

This research is a descriptive qualitative study (9) aimed at exploring in depth the real-life experiences and interactive processes of middle-aged and young patients with coronary heart disease and their spouses during the perioperative period of percutaneous coronary intervention (PCI). Face-to-face semistructured interviews were conducted. For data analysis, this study employed directed content analysis (10). The aim was to objectively and systematically summarize and describe the dyadic coping experiences of middle-aged and young PCI patients and their spouses within the framework of the theory of dyadic illness management (TDIM). The entire research process and report strictly adhered to the COREQ reporting guidelines for qualitative research (11).

### 2.1 Study population

Through purposive sampling, middle-aged and young patients who underwent PCI for coronary heart disease and their spouses were selected as study subjects from the cardiology department of a tertiary-level Class A hospital in Guangxi from August–September 2025. Initially, 25 pairs of patients undergoing PCI and their spouses were invited to participate in the inpatient ward. Among them, 21 pairs of invited patients and their spouses agreed to participate in the study (four couples declined to be interviewed: two because of privacy concerns and two because of poor health). The data from two pilot interviews were intended to assess the scientific validity, rationality and feasibility of the interview guide rather than to collect formal data. Consequently, their content was not included in the final study results. A total of 19 couples were ultimately included in the study. No significant differences in demographic characteristics between couples who did not participate, those who agreed to participate, and those who were excluded were observed. After 16 interviews were completed, only supplementary cases of existing codes were identified; no new codes or themes emerged, indicating that data collection had reached saturation. Three additional interviews were subsequently conducted to confirm that no new themes would arise. Consequently, data collection was concluded following the 19th interview.

### 2.2 Inclusion criteria

Patients:

① Met the World Health Organization (WHO) age classification for young and middle-aged adults and China's minimum legal marriage age (male patients aged 22–59 years and female patients aged 20–59 years) and were married with a surviving spouse; ② met the American Heart Association (AHA) 2023 diagnostic criteria for coronary artery disease (12); ③ underwent PCI for the first time; ④ were conscious and capable of accurate verbal expression; and ⑤ voluntarily participated and signed an informed consent form.

Spouses:

① Were conscious and capable of accurate verbal expression and ② voluntarily participated and signed an informed consent form.

### 2.3 Exclusion criteria

Patients:

① Had a history of psychiatric or psychological disorders; ② had a presence of other severe somatic diseases; and ③ experienced a sudden deterioration of their medical condition during the interview.

Spouses:

① had a history of psychiatric or psychological disorders and ② had a presence of other severe somatic diseases.

### 2.4 Data collection method

This study was conducted collaboratively by two female Master's in Nursing students (Interviewers A and B). Interviewer A acted as the lead interviewer, guiding the conversation according to the interview guide and employing experiential and situational probing strategies. Interviewer B acted as an observer, simultaneously recording nonverbal cues (such as facial expressions and body language) and environmental details and supplementing any omissions after the interview. Both interviewers possessed undergraduate degrees in nursing and clinical experience in cardiovascular medicine. They were familiar with perioperative nursing procedures for PCI and underwent a week-long systematic training program prior to the formal interviews, covering semistructured interview techniques, probing strategies and research ethics. Given that clinical backgrounds may lead to preconceptions (such as the assumption that spousal caregiving is subordinate), the study ensured objectivity through two measures: First, the interviewers wrote reflective notes within 24 h of each interview and recorded nonverbal details (such as sighs) and their own subjective reflections. Second, they conducted regular peer debriefings with a research supervisor who had not participated in the interviews, jointly reviewing potential leading questions or subjective biases to ensure the rigor of data collection. The two interviewers conducted face-to-face in-depth semistructured interviews with the participating couples. Prior to the interview, Interviewer A explained the purpose, significance and procedure of the study to the couple in detail. The interview commenced after informed consent was obtained.

This study employed a joint couple interview format (13), which was primarily based on the theory of dyadic illness management and focused on the interactive process. This approach emphasizes the dynamic nature of the couple as a unit facing the pressures of illness together. Joint interviews enable the real-time capture of nonverbal cues (such as eye contact and physical touch), immediate emotional feedback, and the process of jointly constructing narratives—aspects that are difficult to achieve through individual interviews. Furthermore, given that couples' memories of coping with illness are often intertwined, joint interviews allow both partners to complement and correct one another during the dialogue, thereby helping to reconstruct a more complete picture of their coping strategies. Although individual interviews can delve more deeply into an individual's private experiences, this study aims to prioritize the exploration of the dyadic interaction characteristics between spouses. Consequently, balancing the authenticity of the interaction against individual privacy, this study strategically opted for the joint interview format, which allows for direct observation of the interaction process.

The interviews were conducted in the inpatient ward, avoiding treatment or mealtimes to maintain a quiet and private environment. Each couple underwent a single in-depth interview. The duration of each session was dynamically adjusted according to the participants' tolerance and the depth of the dialogue (averaging approximately 30 min, ranging from 25 to 45 min). The sample size was determined strictly in accordance with the principle of data saturation. Recruitment ceased when data analysis no longer revealed new themes or codes, thereby ensuring the adequacy of the data and the depth of the research.

### 2.5 Determination of the interview outline

In accordance with the research objectives and drawing on the theory of dyadic illness management (6) and a review of the literature on dyadic coping, a preliminary interview outline was developed. This preliminary guide was used to conduct pilot interviews with two pairs of middle-aged and young patients who had undergone PCI for coronary heart disease and their spouses. It was found that some questions in the preliminary outline failed to adequately distinguish between the individual experiences of patients and their spouses and that the exploration of the impact of their interactions was not sufficiently in-depth. Consequently, the research team revised the draft: On the one hand, the questions were clearly divided into patient and spouse versions to ensure the independence of the questioning perspectives. On the other hand, specific items regarding mutual influence and joint coping were refined. Following these adjustments, a formal interview outline was finalized, as detailed in Table 1.

**Table 1:** Interview guide.

Patient
1. When you learned that you had coronary artery disease and needed to undergo PCI, what were your thoughts?
2. After you underwent PCI, how did your spouse change? How did this affect you?
3. How did you cope with this illness?
4. What are your and your spouse’s future plans regarding the treatment of this disease?
Spouse
1. When you learned that your spouse had coronary heart disease and needed to undergo PCI, what were your thoughts?
2. What changes can you expect after your spouse undergoes PCI?
3. Your spouse has coronary heart disease and underwent PCI. How did you cope with this?
4. What are your and your spouse’s future plans regarding the treatment of this disease?

### 2.6 Data analysis

The interview recordings were transcribed verbatim by two graduate nursing students within 24 h of each interview. The researchers cross-checked the transcriptions against the recordings to ensure accuracy. The researchers also took notes to interpret the data and facilitate organization and reflection on existing information. All interview records were anonymized. All the data—including audio files, transcripts, handwritten notes, and informed consent forms—were stored in locked drawers and password-protected computer files. All textual materials were imported into NVivo 15.0 qualitative analysis software for management. Directed content analysis (10) was employed to analyze and code the text content. In this study, the original interviewers, A and B, jointly served as coders. The specific coding steps were as follows. (1) In open coding, the text was read repeatedly, key semantic units were extracted word by word, and preliminary conceptual labels were assigned to the data. (2) In axial coding, the attributes and relationships between codes were compared, similar codes were clustered into subcategories, and logical connections between concepts were established. (3) In selective coding, the subcategories were further refined, core categories were identified, and a thematic framework was constructed. To ensure the reliability of the results, the two coders conducted independent coding and then compared their findings. For coding discrepancies, they first discussed the matter by reviewing the original audio recordings and Coders B's observation notes, using the different perspectives held by both parties to corroborate their interpretations. If consensus could not be reached, a research supervisor who had not participated in the interviews was invited to act as a third-party arbitrator until agreement was reached.

### 2.7 Ethical requirements

This study adhered to the ethical standards outlined in the 1964 Declaration of Helsinki and its subsequent revisions. Prior to the interviews, the participants signed written informed consent forms and verbally reconfirmed their consent during the interviews. All participants signed informed consent forms. These forms explicitly stated that responses would be anonymized after data collection and that the participants retained the right to withdraw from the study at any time before data publication. This study received ethical approval from the Medical Ethics Committee of the Second Affiliated Hospital of Guangxi Medical University [ethics approval number: 2025-KYL(043)].

### 2.8 Rigor

This study strictly adhered to the ethical standards and methodological requirements of qualitative research. A strategy to ensure rigor was developed across four dimensions—credibility, reliability, validity and generalizability—to guarantee the scientific rigor of the research process and the reliability of the findings. To enhance the credibility of the study, a strategy involving member validation and peer feedback was adopted: Following the formation of a thematic framework through preliminary analysis, the researcher fed back key findings and interpretations to a selected group of research participants (three couples), inviting them to confirm whether the research conclusions aligned with their actual experiences and making fine-tuning adjustments on the basis of their feedback. Simultaneously, the research team held regular seminars with research supervisors and project members who had not participated in the interviews, utilizing peer perspectives to scrutinize potential biases in the research process and to ensure that the interpretation of the data was objective and reasonable. To ensure the reliability of the research process, this study established interview procedures, probing strategies, transcription guidelines and coding standards. All interview recordings, transcribed texts, reflection notes and coding trees were systematically managed using NVivo 15.0 software, creating a clear audit trail to ensure that the research process was traceable and subject to review. In terms of verifiability, this study implemented a triangulation strategy, including researcher triangulation (where two interviewers coded the data independently and cross-checked their findings) and data triangulation (cross-referencing interview transcripts with nonverbal information recorded by observers and reflective notes), thereby minimizing the influence of individual researcher subjectivity on the results. Simultaneously, the interviewers wrote reflective notes after each interview, explicitly documenting any preconceptions that might arise from their own backgrounds, and they actively suspended personal judgments during the analysis. To enhance the generalizability of the findings, this study provides a detailed description of the participants' background and strictly adheres to the COREQ reporting guidelines, reporting in detail the process of developing and revising the interview guide, as well as the specific steps of data collection and analysis. This enables readers to assess the similarity between the context of this study and their own context of interest, thereby determining whether to generalize the conclusions of this study.

## 3 Results

General information regarding the patients and their spouses is detailed in Tables 2, 3.

**Table 2:** Patient characteristics (*n* = 19).

Patient ID	Age	Sex	Occupation	Residence	Educational background	Medical expense payment methods	Monthly income	Number of stents	Comorbidities^a^
A	37	Male	Farmer	Rural	High school	New Rural Cooperative Medical Scheme	3,000–5,000 yuan	1	Combined with two diseases
B	58	Male	Farmer	Rural	Junior high school or below	Urban Employee Medical Insurance/Urban Resident Medical Insurance	＜3000 yuan	2–3	Combined with three or more diseases
C	54	Male	Retiree	Township	High school	Urban Employee Medical Insurance/Urban Resident Medical Insurance	3,000–5,000 yuan	2–3	Combined with one disease
D	52	Male	Farmer	Rural	Junior high school or below	New Rural Cooperative Medical Scheme	3,000–5,000 yuan	1	None
E	55	Male	Self-employed	Rural	Junior high school or below	New Rural Cooperative Medical Scheme	＜3000 yuan	4–5	Combined with one disease
F	56	Male	Public institution employee	Township	Bachelor's degree	Urban Employee Medical Insurance/Urban Resident Medical Insurance	3,000–5,000 yuan	2–3	Combined with three or more diseases
G	57	Male	Public institution employee	Township	Associate degree	Urban Employee Medical Insurance/Urban Resident Medical Insurance	3,000–5,000 yuan	1	None
H	53	Male	Self-employed	Township	Junior high school or below	New Rural Cooperative Medical Scheme	＜3000 yuan	1	Combined with three or more diseases
I	47	Female	Enterprise employee	City	Junior high school or below	Urban Employee Medical Insurance/Urban Resident Medical Insurance	＜3000 yuan	1	Combined with one disease
J	58	Male	Farmer	City	Junior high school or below	New Rural Cooperative Medical Scheme	＜3000 yuan	2–3	Combined with three or more diseases
K	47	Male	Self-employed	City	Associate degree	New Rural Cooperative Medical Scheme	3,000–5,000 yuan	4–5	Combined with one disease
L	55	Female	Self-employed	City	Junior high school or below	New Rural Cooperative Medical Scheme	＜3000 yuan	2–3	Combined with one disease
M	48	Male	Self-employed	Rural	Junior high school or below	New Rural Cooperative Medical Scheme	＜3000 yuan	1	Combined with one disease
N	48	Female	Farmer	Rural	Junior high school or below	New Rural Cooperative Medical Scheme	3,000–5,000 yuan	2–3	Combined with one disease
O	40	Male	Self-employed	City	Junior high school or below	Urban Employee Medical Insurance/Urban Resident Medical Insurance	3,000–5,000 yuan	2–3	None
P	56	Male	Farmer	Rural	Junior high school or below	New Rural Cooperative Medical Scheme	＜3000 yuan	4–5	Combined with three or more diseases
Q	40	Male	Farmer	Rural	Junior high school or below	New Rural Cooperative Medical Scheme	＜3000 yuan	1	Combined with three or more diseases
R	58	Male	Public institution employee	Township	Junior high school or below	New Rural Cooperative Medical Scheme	3,000–5,000 yuan	4–5	None
S	59	Female	Farmer	Rural	Junior high school or below	New Rural Cooperative Medical Scheme	＜3000 yuan	2–3	Combined with three or more diseases

a Comorbidities: This refers to other independent conditions (such as hypertension and diabetes) that coexist with coronary heart disease at the time of diagnosis.

**Table 3:** General information on spouses (*n* = 19).

Patient ID	Age	Sex	Occupation	Residence	Educational background	Monthly income
A1	42	Female	Self-employed	Rural	Junior high school or below	3,000–5,000 yuan
B1	54	Female	Self-employed	Rural	Junior high school or below	＜3000 yuan
C1	51	Female	Retiree	Township	Associate degree	＜3000 yuan
D1	52	Female	Self-employed	Rural	Junior high school or below	＜3000 yuan
E1	55	Female	Self-employed	Rural	Junior high school or below	＜3000 yuan
F1	49	Female	Public institution employee	Township	Bachelor's degree	3,000–5,000 yuan
G1	55	Female	Public institution employee	Township	High school	3,000–5,000 yuan
H1	53	Female	Self-employed	Township	Junior high school or below	＜3000 yuan
I1	50	Male	Self-employed	City	Junior high school or below	>5000 yuan
J1	58	Female	Self-employed	City	Junior high school or below	＜3000 yuan
K1	47	Female	Self-employed	City	High school	3,000–5,000 yuan
L1	59	Male	Self-employed	City	Junior high school or below	＜3000 yuan
M1	45	Female	Self-employed	Rural	Junior high school or below	＜3000 yuan
N1	52	Male	Worker	Rural	Junior high school or below	3,000–5,000 yuan
O1	38	Female	Self-employed	City	Junior high school or below	＜3000 yuan
P1	57	Female	Farmer	Rural	Junior high school or below	＜3000 yuan
Q1	40	Female	Farmer	Rural	Junior high school or below	＜3000 yuan
R1	60	Female	Self-employed	Township	Junior high school or below	＜3000 yuan
S1	67	Male	Farmer	Rural	Junior high school or below	＜3000 yuan

Through in-depth interviews with patients who had undergone PCI and their spouses, this study utilized an analytical framework based on the dyadic process model of illness management. Starting from the interactive chain of dyadic assessment → dyadic behavior → dyadic response → dyadic planning, this study revealed the characteristics of the process through which couples jointly cope with illness during the critical period spanning from the decision-making stage for stent implantation to the early postoperative rehabilitation phase. Ultimately, five themes and ten subthemes were identified, as detailed below:

### 3.1 A: different coping tendencies during the stent decision period

Diagnosis and treatment recommendations for stent implantation serve as the initial shock of the illness; the degree of agreement between the couple regarding their shared assessment of this event influences their subsequent coping strategies. The degree of agreement depends on the cognitive resources available within the family.

#### 3.1.1 A1: rational coping through cognitive empowerment

When at least one partner has prior experience (such as a relative who has undergone stent placement) or a family member with a medical background is able to provide expert explanations, both partners are able to quickly reach a shared, positive assessment, viewing the stent as an effective solution. This perspective reduces anxiety caused by uncertainty and leads to rational cooperation.

“I wanted to get the stent so I could recover faster and avoid enduring too much suffering. Before the procedure, I felt terrible—worse than I'd imagined. Since my mother had already undergone stenting once, I understood it could resolve my issue and fully supported the decision.” (I)

“I didn't think much of it at the time. I considered it quite normal. My daughter studies medicine, and she reassured me it was fine. Yes, just get the stent placed. If they tell you to do it, do it. Keep a level head.” (D)

“If it needs to be done, just do it. I didn't feel anything about it. His (the patient's) mother had a stent placed. Someone in the family had it done, so I didn't think much of it.” (P1)

#### 3.1.2 A2: information deficit triggers resistance

When both partners lack knowledge about the condition, they may react to the suggestion of stenting with shock, anxiety or even resistance. As they are unable to make an accurate assessment of benefits and risks, it is difficult for the couple to reach a shared decision, resulting in a passive, hesitant or contradictory response.

“When the doctor mentioned surgery, I said, ’Surgery? If it's surgery, I won't do it. Forget it.’ Then, the doctor said it didn't require surgery. If it did, I wouldn't have done it. I was definitely nervous.” (M)

“At the time, I just hoped they wouldn't have to place it if possible. I was definitely nervous and anxious. I thought it was just an examination, but then they said they needed to open it up promptly, or they'd have to insert another tube later.” (R)

“I was a bit anxious, still a bit nervous, anxious.” (D1)

“I was panicking, didn't know what to do. The doctor said to implant it, so implant it. If it can be done, do it. If it can be treated, treat it. I told his family, and they came too.” (Q1)

### 3.2 B. Dual Financial Pressures Exacerbate the Psychological Burden

Stent implantation not only entails direct medical costs but also triggers a chain reaction of financial hardship within the family. Both spouses are forced to reallocate their limited financial and caregiving resources, creating a process of dyadic resource reallocation characterized by implicit negotiations involving sacrifice and compensation.

#### 3.2.1 B1: concerns over medical costs

Medical expenses are a direct source of concern for both spouses. Given insufficient health insurance coverage and limited household savings, both partners display marked anxiety, which is often exacerbated by their interactions with one another.

“Of course you think about it—how much will the stent cost? Oh, what about all the other expenses?” (H)

“It's stressful—the money needed. Since we don't have money, anxiety is inevitable. The financial pressure is immense.” (J)

#### 3.2.2 B2: increased indirect family burdens

To ensure that patients receive postoperative care, some spouses are forced to resign, reduce their working hours or turn down job opportunities, which directly leads to a reduction in household income. At the same time, additional expenses such as transport and food during the caregiving process further exacerbate the financial burden. The combined risk of “treatment-induced poverty” and “care-induced poverty” causes the burden of illness to spread from the individual level to the entire household's financial system.

“(I) just quit my job. I couldn't handle it all—caring for him plus two kids. Business at the shop isn't good now either; we're often losing money.” (A1)

“We have no money. We're borrowing from relatives now. Jobs are hard to find, and work is unstable.” (Q1)

### 3.3 C: conflictual spousal interactions

Postsurgery spousal interactions exhibit distinct dual characteristics. While spouses often attribute the illness to the patient's past unhealthy habits and express blame, they still hold the primary caregiving responsibilities in practice.

#### 3.3.1 C1: attribution and blame in emotional venting

During the recovery phase, some spouses linked the patient's illness to unhealthy lifestyle choices (e.g., smoking and poor dietary habits), expressing emotions through blame and complaints. This expression fundamentally served as an outlet for the spouse's anxiety, fear, and helplessness in the face of sudden illness rather than mere accusation.

“My wife scolded me too, saying I didn't quit smoking even though she told me to.” (A)

“She told me to quit smoking, but I didn't. So, even after getting the stent, she still complained about me.” (D)

“It's his (the patient's) own responsibility. It's a done deal now. I won't say more.” (F1)

#### 3.3.2 C2: resolute support in action

Despite emotional outbursts of blame, spouses demonstrated strong responsibility through concrete actions: voluntarily taking on household chores, quitting work to provide full-time care, offering emotional comfort, and accompanying the patient to follow-up appointments. This steadfast support through action became a crucial safeguard for the patient's postoperative recovery and embodied the core bond within their intimate relationship.

“I quit my job to care for him full-time and be by his side. We also have two children who need care, and we run two small shops that require our attention.” (A1)

“She wanted to come stay with me, but I told her not to. She works late shifts, and by the time she finishes, it's past 8 PM. I used to handle all the housework, but now she does it.” (D)

“I give (the patient) all the support—psychologically, hoping he can overcome the illness—and I keep accompanying him to appointments.” (F1)

### 3.4 D: patients' perceptions of spousal support vary

Patients' understanding of their spouses' caregiving behaviors reveals two distinct cognitive frameworks that reflect different marital interaction dynamics.

#### 3.4.1 D1: support perceived as a marital norm

Some patients view their spouse's assistance as a natural component of marriage and lack emotional reciprocity. While this mindset maintains the stability of the current caregiving system, it may suppress emotional exchange over time, potentially sowing seeds of relational tension.

“I wouldn't say I'm particularly moved by it. It just feels normal—spouses helping each other is natural.” (A)

“I just think it's expected—helping each other out.” (C)

“(The help) is what should be done.” (K)

#### 3.4.2 D2: support transformed into emotional reinforcement

Another group of patients deeply appreciate their spouse's efforts, viewing them as an opportunity to enhance marital quality. This positive perception not only strengthens the patient's confidence in recovery but also reinforces the emotional bond between spouses.

“He became more attentive. Like that night after my surgery, when I needed medication and a pump (for intravenous infusion). He stayed up all night with me. The next morning, after the medical staff removed the pump, I told him to go back to handle his company matters. But he waited until six or seven in the evening before heading home. I was deeply touched.” (I)

“She cooks at home and handles everything. If I feel unwell, she urges me to see a doctor. She wants me to be healthy and recover soon—whether I can work or not doesn't matter. It's touching that she cares so deeply for me.” (J)

“She treats me even better than before—giving more emotional encouragement and support. Everything is good, in every way. It's touching and comforting.” (N)

### 3.5 E: lack of clear understanding regarding long-term disease management

Long-term health management is required following PCI. However, most couples fail to establish a shared plan of action and lack a long-term management strategy involving joint discussion, a division of responsibilities and mutual commitment.

#### 3.5.1 E1: absence of long-term disease management plans

Some respondents adopted an ambiguous or evasive stance regarding follow-up management. The couple had never held a systematic discussion regarding the supervision of medication after discharge, the division of responsibilities for follow-up appointments, or adjustments to their lifestyle. This collective avoidance reflects a short-sighted view of the future under a crisis response model, and it is also a direct consequence of the healthcare system failing to trigger a dyadic planning approach.

“No plan. We'll take it one step at a time.” (B)

“Nothing special. Just get better and go home.” (L1)

“Don't know. Never thought about it.” (P)

#### 3.5.2 E2: developing basic disease management awareness

Only a few couples proposed simplified self-management strategies. Although unsystematic, these initial insights serve as entry points for nursing interventions.

“Watching diet, exercising, less oil and salt, staying positive—and reducing anxiety.” (A1)

“Just take meds, eat light, and exercise regularly.” (G)

## 4 Discussion

This study revealed that families with prior experience or a medical background were able to rapidly form a consistent and positive assessment, which is highly consistent with Bandura's social cognitive theory (14) and the concept of dyadic assessment within the theory of dyadic illness management (6). Bandura's social cognitive theory suggests that an individual's sense of self-efficacy not only stems from direct experience but also can be enhanced by observing the successful experiences of others. The concept of dyadic evaluation within dyadic disease management theory emphasizes that spouses, acting as a management team, must jointly acquire, share and integrate health information to form a consistent judgment regarding the threat posed by the illness. However, the majority of patients and their spouses in this study's sample had low levels of education and hailed from rural or township areas. Their insufficient health literacy often led to confused or divided assessments. This finding suggests that the dyadic assessment within the theory of dyadic illness management is not merely an information-processing process at the individual level. Rather, it is an interactive process involving the dynamic sharing of cognitive resources between spouses. In rural households with low educational attainment, family members rarely possess a medical background, and few relatives or friends have experience with PCI treatment. The scarcity of vicarious experience makes it difficult for couples to increase their self-efficacy by observing others' successful experiences, forcing them to rely entirely on the one-way information provided by healthcare professionals. Consequently, the quality of dyadic assessment depends more on proactive intervention from the external healthcare system than on the family's internal information resources. Therefore, clinical nursing staff should proactively assess the cognitive resources of both spouses during preoperative communication, adopt visual and contextualized communication strategies (such as animated demonstrations or displays of heart models), and encourage family members with similar treatment experiences to participate in preoperative discussions, thereby leveraging peer education to enhance vicarious efficacy (15).

Economic toxicity (16) refers to the negative impact that the financial burden of illness has on patients and their families during the course of treatment. In this study, it manifests as the combined effect of direct medical costs and the impoverishment resulting from indirect caregiving. The theory of dyadic illness management (6) focuses primarily on disease management behaviors themselves, with little mention of structural pressures such as economic factors. This study reveals that economic toxicity should be regarded as a key external moderating variable in dyadic coping. In low-income rural households, economic toxicity alters the rules governing the allocation of resources between spouses: One partner is forced to resign, plunging the family into an unbalanced dyadic structure characterized by one-sided sacrifice and hidden debt. Patients often find themselves unable to express gratitude because of feelings of guilt, which further inhibits the positive feedback loop. While this phenomenon may be relatively rare in urban middle- and high-income households, it serves as a reminder that economic toxicity is a structural variable that cannot be overlooked when understanding dyadic coping mechanisms in low-resource contexts. The findings above suggest that economic pressure is not only an individual burden on the patient but also a systemic force that reshapes resource allocation and interaction patterns between spouses. Therefore, in clinical nursing practice, it is necessary to incorporate family economic circumstances into routine assessments of dyadic coping while paying attention to latent emotional conflicts (such as guilt and self-blame) that arise from financial burdens and their inhibitory effect on spousal communication. Furthermore, the participants' widespread concerns regarding medical costs highlight a gap in information support. This gap suggests that healthcare professionals should prioritize transparent communication regarding health insurance reimbursement policies and in-hospital financial assistance channels during perioperative communication to alleviate anxiety exacerbated by information asymmetry (17).

This study revealed that interactions between patients who have undergone PCI for coronary heart disease and their spouses are characterized by a degree of contradiction: Spouses often vent their anxiety and feelings of helplessness in the face of sudden illness through verbal attributions. However, at the behavioral level, they continue to actively assume caregiving responsibilities, such as accompanying the patient to follow-up appointments, monitoring medication intake, and adjusting their diet. This phenomenon closely aligns with Bodenmann's dyadic coping theory (18), which posits that partners may express negative emotions while simultaneously providing substantive support in stressful situations, reflecting the intertwining of emotional complexity and responsibility-taking within intimate relationships. Notably, the interactions observed in this study occurred in the presence of both partners. Although we captured verbal reproaches, it can be inferred that in one-on-one situations, the care burden expressed by the spouse or the deep-seated anxiety expressed by the patient may be even more intense and complex. However, if such a communication pattern of “verbal complaints but practical support” remains unrecognized and unaddressed over the long term, the underlying emotional tension may gradually accumulate, evolving into chronic marital conflict and even undermining the spouse's willingness to provide care and the patient's confidence in recovery. Based on the theory of dyadic illness management (6), nursing staff should introduce structured couple communication interventions (19) to transform blame into the expression of feelings and needs. Notably, within the cultural context of rural China, the direct expression of emotional care is often viewed as overly sentimental, whereas blame and complaints are instead regarded as acceptable forms of care. In this study, the spouses’ attributional blame was not merely a result of poor communication; rather, it was a culturally embedded emotion-focused coping strategy. This finding suggests that dyadic intervention programs should not simply replicate Western models of positive communication; instead, they should respect local cultural expression habits. While retaining the strengths of practical support, such programs should gradually guide couples to identify the emotional needs underlying their blame.

This study revealed variations in how patients perceive their spouse's caregiving support. Some patients take their spouse's contributions for granted. While doing so may maintain superficial family stability in the short term, a lack of emotional feedback risks diminishing the spouse's caregiving motivation and psychological energy. Conversely, patients who express gratitude and actively acknowledge their partner's efforts are more likely to establish a positive “support–response–resupport” cycle with their spouse, fostering mutual psychological adaptation and relationship resilience. These findings suggest that care interventions should focus on enhancing “emotional visibility” in spousal interactions, ensuring that each partner's contributions are acknowledged, articulated, and valued. Specifically, patients can be encouraged to express gratitude verbally or through actions, while spouses are guided to openly share caregiving pressures, expectations, and emotional needs, thereby breaking the imbalance of one-sided giving (20).

Our findings reveal that most patients who undergo PCI and their spouses lack a clear understanding of long-term disease management, which is consistent with the findings of a previous study by Zhu (21). The theory of dyadic illness management (6) emphasizes that dyadic planning is a core component of effective disease management. This study revealed that this component is generally lacking in clinical practice. Limited by educational background and living conditions, the majority of participants had restricted access to health information, making it difficult to obtain disease-related knowledge through diverse channels. Furthermore, shortages of nursing staff in Chinese medical institutions prevent nurses from providing systematic, in-depth health education to patients and their spouses. Compounded by the rapid recovery and short hospitalization duration of patients who undergo PCI, delivering joint health guidance to couples within the limited inpatient period presents practical challenges (22). Therefore, nursing staff should prioritize middle-aged and young patients who undergo PCI with lower educational attainment and limited information access, along with their spouses. The establishment of a postdischarge continuity-of-care intervention model is recommended. The use of information technology tools such as online platforms, mobile applications, and telemedicine can enable remote management and support for the home rehabilitation of patients and their spouses (23–25).

Notably, the findings of this study are contingent upon a specific sociocultural context. The characteristics exhibited by the majority of families in the sample—namely, low health literacy, high economic vulnerability, traditional gender roles and restrained emotional expression—may have collectively shaped the cognitive differentiation during the decision-making phase, the amplifying effect of economic pressures and the contradictory pattern of verbal complaints coupled with practical support. Consequently, caution is advised when generalizing the conclusions of this study to families in urban areas, those with higher educational attainment or higher incomes, or those from different cultural backgrounds, as the underlying dynamics of this dyadic coping strategy may vary.

### 4.1 Limitations

This study has certain limitations. Although the joint couple interview method employed in this study is effective in capturing the interactive process, it is also subject to specific potential biases. First, influenced by the Chinese cultural emphasis on family harmony and social expectations, the respondents may have tended to engage in self-censorship when their partner was present, reporting only the positive aspects of sensitive topics such as blame, conflict or the burden of care while omitting the negative, resulting in an underrepresentation of negative emotions and conflicts. Second, disparities in the power to speak between spouses may have led to one party dominating the narrative, thereby suppressing the authentic voice of the weaker party. Furthermore, the joint interviews tended to encourage both parties to construct a consensus narrative, masking genuine internal disagreements. To mitigate such biases, this study implemented several remedial measures: The interviewer paid close attention to nonverbal cues (such as sighs and averted eye contact) to uncover genuine emotions. Member validation was used to confirm the existence of contradictory interactions, such as verbal complaints accompanied by supportive actions. Triangulation was achieved through the use of field notes and reflective notes. For future research aiming to explore individuals' hidden psychological states (such as feelings of guilt or depression) in greater depth, adopting a hybrid interview model that combines joint and individual sessions to obtain a more multidimensional perspective is recommended. Second, the sample primarily consisted of middle-aged and young male patients from rural areas and their spouses. While this sample composition aligns with the epidemiological pattern of higher CHD incidence among men (26), it exhibits limited sex and regional representativeness. The experiences of female patients and urban residents were not sufficiently incorporated. Their health literacy, medical insurance coverage, information access channels, and family communication patterns may differ significantly. Consequently, caution should be exercised when generalizing the findings of this study to other populations.

### 4.2 Strengths and innovative aspects

Based on the theory of dyadic illness management, this study systematically analyzes the dual coping dynamics of middle-aged and young patients with coronary heart disease and their spouses within the Chinese cultural context, providing a reference for qualitative research in this field within China.This study proposes a family-centered dyadic collaborative nursing intervention program that integrates health literacy enhancement, identification of economic toxicity, couple communication interventions, and information-based continuity of care, thereby providing practical, localized strategies for clinical nursing practice.

## 5 Conclusion

Based on dyadic disease management theory, in this study, in-depth interviews were conducted with middle-aged and young patients with CHD and their spouses during the PCI perioperative period. Five themes emerged: differing coping tendencies during stent decision-making, a heightened psychological burden from dyadic economic pressures, contradictory spousal interactions, divergent perceptions of spousal support among patients, and a lack of clear understanding of long-term disease management. These findings indicate that spouses play an indispensable role in providing care and support during the PCI perioperative period. Therefore, it is recommended that clinical nursing practices integrate spouses into the disease management system for patients with CHD. Doing so should involve establishing a dyadic-collaborative nursing intervention model tailored to the local cultural context and centered on the patient–spouse unit, thereby enhancing disease management outcomes and overall patient recovery quality.

## Data Availability

The original interview transcripts and coding data presented in this study are not publicly available because of privacy and ethical restrictions. Further inquiries can be directed to the corresponding author.
